# Epigenetic age acceleration is a distinctive trait of epithelioid sarcoma with potential therapeutic implications

**DOI:** 10.1007/s11357-024-01156-6

**Published:** 2024-06-16

**Authors:** Simon Haefliger, Olga Chervova, Christopher Davies, Chet Loh, Roberto Tirabosco, Fernanda Amary, Nischalan Pillay, Steve Horvath, Stephan Beck, Adrienne M. Flanagan, Iben Lyskjær

**Affiliations:** 1 Research Department of Pathology, University College London, UCL Cancer Institute, London, UK; 2 Bone Tumor Reference Centre, Institute of Medical Genetics and Pathology, University Hospital Basel, University of Basel, Basel, Switzerland; 3 Department of Histopathology, Royal National Orthopaedic Hospital, Stanmore, London, UK; 4 Medical Genomics Research Group, University College London, UCL Cancer Institute, London, UK; 5 Altos Labs, Cambridge Institute of Science, Cambridge, UK; 6 Department of Molecular Medicine, Aarhus University Hospital, Aarhus, Denmark

**Keywords:** DNA methylation age, Epithelioid sarcoma, *SMARCB1*, PRC2 complex, Anti-aging therapy

## Abstract

Recently, DNA methylation clocks have been proven to be precise age predictors, and the application of these clocks in cancer tissue has revealed a global age acceleration in a majority of cancer subtypes when compared to normal tissue from the same individual. The polycomb repressor complex 2 plays a pivotal role in the aging process, and its targets have been shown to be enriched in CpG sites that gain methylation with age. This complex is further regulated by the chromatin remodeling complex SWItch/Sucrose Non-Fermentable and its core subunit, notably the tumor suppressor gene *SMARCB1*, which under physiological conditions inhibits the activity of the polycomb repressor complex 2. Hence, the loss of function of core members of the SWItch/sucrose non-fermentable complex, such as the tumor suppressor gene *SMARCB1*, results in increased activity of polycomb repressor complex 2 and interferes with the aging process. *SMARCB1*-deficient neoplasms represent a family of rare tumors, including amongst others malignant rhabdoid tumors, atypical teratoid and rhabdoid tumors, and epithelioid sarcomas. As aging pathways have recently been proposed as therapeutic targets for various cancer types, these tumors represent candidates for testing such treatments. Here, by deriving epigenetic age scores from more than 1000 tumor samples, we identified epigenetic age acceleration as a hallmark feature of epithelioid sarcoma. This observation highlights the potential of targeting aging pathways as an innovative treatment approach for patients with epithelioid sarcoma.

**Supplementary Information:**

The online version contains supplementary material available at 10.1007/s11357-024-01156-6.

## Introduction

Age is a major risk factor for cancer, and there is considerable overlap between the biological processes of aging and cancer [1]. Numerous studies have demonstrated that specific CpG sites in DNA are epigenetically modified in an age-dependent manner, and epigenetic clocks assessed through DNA methylation (DNAm) levels in normal tissues and blood can be used as accurate surrogate markers for chronological age (CA) [2]. The determination of DNAm age based on tissue samples has been performed for a variety of cancers and has shown a global age acceleration in cancer samples compared to the corresponding normal tissue. Nevertheless, these changes are variable across cancer types, and age deceleration has also been observed in some cancers [2]. Among the key pathways altered in the process of aging are downstream targets of the polycomb repressive complex 2 (PRC2), which are enriched in CpG sites that acquire age-related methylation [3, 4]. The chromatin remodeling complex SWItch/sucrose non-fermentable (SWI/SNF) is a regulator of PRC2, which in its physiological state exerts an inhibitory action on PRC2 [5]. Hence, the loss of function of core members of the SWI/SNF complex, such as the tumor suppressor gene *SMARCB1*, results in increased PRC2 activity and interferes with the aging process. Indeed, while PRC2 primarily mediates histone modifications (H3K27me3), it also interacts with the DNA methylation machinery by regulating DNA methylation levels of CpG sites involved in aging [6]. Specifically, aging has been shown to correlate with increased methylation levels at PRC2-related CpGs [7–9]. While a comprehensive description of the connection between DNA methylation and PRC2 exists, the underlying mechanistic link is incompletely understood [10–12].

As aging pathways have recently been proposed as potential therapeutic targets for various cancer types, *SMARCB1*-deficient tumors represent candidates for testing such treatments. These neoplasms represent a family of rare tumors, including amongst others malignant rhabdoid tumor (MRT), atypical teratoid and rhabdoid tumor (ATRT), and epithelioid sarcoma (EpS). Although sharing a common molecular hallmark, they differ in anatomical location, age at presentation, and histogenesis [13]. However, they all have limited treatment options. In rare cases, these tumors can have alterations in the *SMARCA4* gene rather than *SMARCB1* [14–16].

Here, we used DNAm data to generate epigenetic age scores and identify opportunities for targeting aging pathways in *SMARCB1*-deficient neoplasms.

## Material and methods

### Patient samples

Patient tissues and data were obtained from the Royal National Orthopaedic Hospital (RNOH, Stanmore, UK) and Royal Orthopaedic Hospital (ROH, Birmingham, UK), which are covered by the Human Tissue Authority license. The use of RNOH samples was approved by the UCL-UCLH Biobank for Health and Disease (project EC17.14). Ethical approval for the biobank was obtained from the Cambridgeshire 2 Research Ethics Service (reference 09/H0308/165). Ethical approval was also given from the ROH Birmingham REF: RTB20-002.

### Samples selection

Fourteen EpS cases were included in the analysis having been identified in the pathology archives using the relevant ICD code. The diagnoses were confirmed as EpS by expert sarcoma pathologists (AMF, RT) who selected cases that fulfilled the WHO diagnostic criteria [17] and were also classified as EpS according to the DKFZ methylation classifier [18]. All cases demonstrated loss of SMARCB1 expression and retained expression of SMARCA4. Supplementary 1, Table 1 provides demographic and clinical data of the cohort.

### Tissue processing and DNA extraction protocol for fresh-frozen (FF) samples

DNA was extracted from tumor samples (FF material) and blood samples. Supplementary 2 provides the details of the DNA extraction protocol.

### DNA methylation protocol

Five hundred nanograms of DNA were bisulfite converted using Zymo EZ DNA methylation gold kit (Zymo Research Corporation, Irvine, CA, USA) and hybridized to the Infinium HumanMethylationEPIC BeadChip arrays (Illumina, San Diego, CA, USA) according to the manufacturer’s recommendations. Methylation profiles of matched blood were also generated for two patients (IDs S00097179 and S00097203).

### Processing of raw methylation data

The generated IDAT files were processed using R (version 4.1.2.) with the package ChAMP (version 2.24.0) [19]. The following filtering parameters were used: probes with a *p*-value > 0.01, probes with < 3 beads in at least 5% of samples per probe, non-CpG probes, all SNP-related probes, multi-hit probes, and probes located on chromosomes X and Y. For the assessment of data quality, the champ.QC function was used. This led to four samples (IDs S00056154, S00056147, S00056153, S00065391) being excluded leaving a total of 14 cases included in the study. BMIQ was used as normalization method. The EPIC array data were converted to a virtual 450 K array for joint normalization and processing of data from both platforms using the combineArrays in the R-package minfi (version 1.40.0) [20]. Batch effects were assessed using the singular value decomposition method. Batch effects related to the source of the data, array type (450 K versus 850 K), and slides were present. These covariates overlapped with the phenotypes (EpS, MRT, and ATRT) as a result of data from different sources with specific characteristics (array type and slides) being combined. These batch effects were inherent to the combined data sets used and could not be adjusted.

### Publicly available DNA methylation data sets

Methylation data from publicly available studies (GSE140686 and GSE70460), including MRT (*n* = 17) and ATRT (*n* = 78), were downloaded and processed as described above [18, 21]. For validation purposes, the processed beta values (*n* = 952) (reference DKFZ dataset) were used for analysis (GSE140686) [18] and included 17 EpS cases. In addition, the processed beta values (*n* = 48) from a cohort of *SMARCA4*-deficient tumors, including ATRT (*n* = 14), MRT (*n* = 6), and small cell carcinoma of the ovary, hypercalcemic-type (SCCOHT) (*n* = 28) were used for additional analysis/validation (GSE161692) [14].

### DNAm age scores

We inferred epigenetic age (EA) scores from three different DNAm epigenetic clocks: the (pan-tissue) clock from Horvath, the Hannum clock, and the PhenoAge clock [2, 22, 23]. In brief, the Horvath clock was developed as a linear combination of 353 CpGs selected by elastic net regression based on DNAm profiles of multiple tissues, and the Hannum blood-based clock was calculated using a linear combination of 71 CpGs to predict chronological age. The PhenoAge clock was constructed as a morbidity and mortality predictor and was calculated based on 513 CpGs. The methylclock library (version 1.0.1) was used to process data in R [24]. As a measure of epigenetic age acceleration (EAA), we considered Δage = EA − CA.

### Statistical analysis

EA and EAA scores were compared between the groups using a two-sided *t*-test with a significance threshold of *p* = 0.01. To account for multiple testing, we used Bonferroni correction. The R-packages ggplot2 (version 3.4.2) and ggbpur (version 0.6.0) were used for statistical analysis and data plotting. The version 4.1.2. of R was used for all statistical analyses.

## Results

### EpS shows the highest EA and EAA across mesenchymal neoplasms

**Fig. 1:**
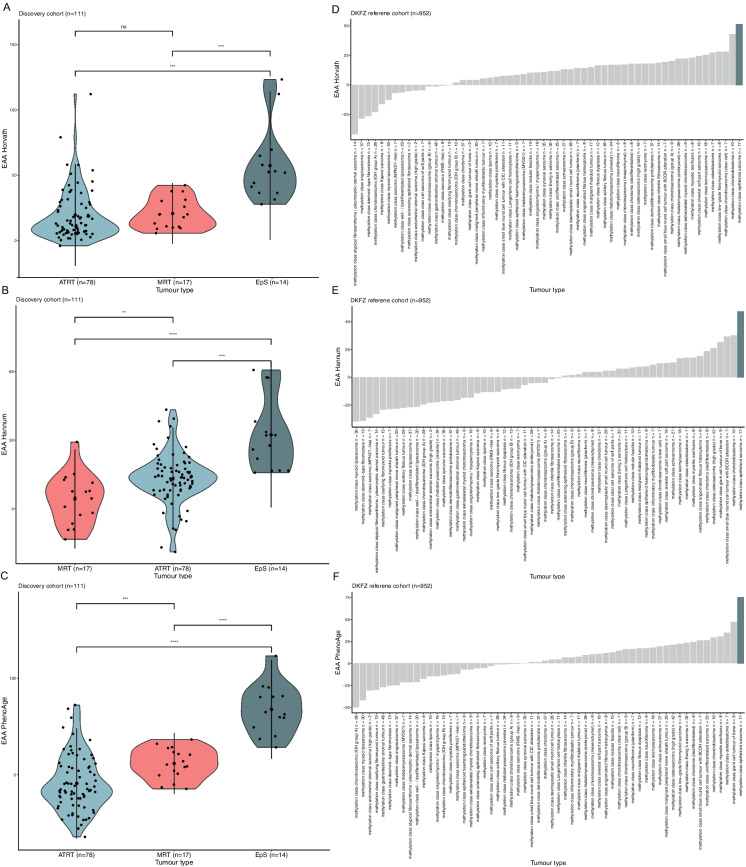
Summary of epigenetic age acceleration (EAA) scores. **A** Violin plots showing scores of EAA across the three SMARCB1-deficient neoplasms, EpS (*n* = 14), MRT (*n* = 17), ATRT (*n* = 78) for the Horvath (**A**), Hannum (**B**), and PhenoAge (**C**) clocks. EpS samples showed the highest EAA score. **D**–**F**. Bar plots showing the mean EAA across 58 types of mesenchymal neoplasms from the DKFZ reference data set (*n* = 952) for the Horvath (**D**), Hannum (**E**), and PhenoAge (**F**) clocks. Mean EAA of EpS samples (*n* = 17) is highlighted and showed the highest value across the data set. The *x*-axis is labeled with the methylation class. Legend: 0.0001 to 0.001 ***; 0.001 to 0.01 **; 0.01 to 0.05 *; ≥ 0.05. *ns* non-significant

Within the *SMARCB1*-deficient neoplasm cohort, EpS showed the highest EA and EAA compared to MRT and ATRT (*p* < 0.01) across all three DNAm clocks (Fig. 1A–C and supplementary 3, Table 2 for all EA and EAA values inferred in the cohort). Within the external validation cohort (DKFZ reference data set), EpS showed the highest EA and EAA when compared with the other 58 mesenchymal neoplasms evaluated (Fig. 1D–F and supplementary 4–5, Fig. 1 and Table 3 for all EA and EAA values inferred in the DKFZ cohort), although this was not found to be significant when compared to all sarcoma subtypes included in the study (*n* = 58) (supplementary 6, Table 4 for all p values (two-sided t-test) comparing the EA and EAA values inferred in the DKFZ cohort).

### Absence of correlation between EA scores from tumor and matched blood samples

**Fig. 2:**
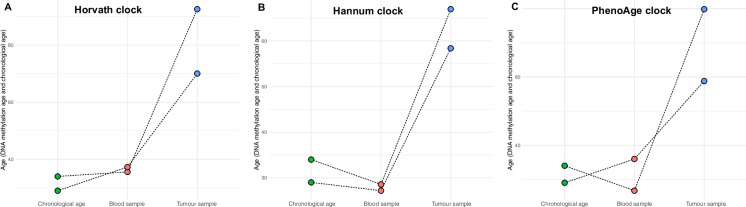
Correlation of EA score of tumor and matched blood samples with chronological age. **A**–**C** Plots showing the difference between the chronological age and matched DNA methylation age in the blood and in the tumor sample for two patients for the Horvath (**A**), Hannum (**B**), and PhenoAge (**C**) clocks

To explore the systemic effect underlying the age acceleration observed in EpS, the matched blood of two cases was analyzed. This showed that the blood EA was close to the patients’ CA (mean Δage = 4.94, − 3.63, − 0.17 years for the Horvath, Hannum clock, and PhenoAge clocks, respectively) in striking contrast to the EA scores of the tumor samples (mean Δage = 49.84, 31.18, 37.81 years for the Horvath, Hannum clock, and PhenoAge clocks, respectively) (Fig. 2A–C).

### EA is higher in EpS compared to SMARCA4-deficient neoplasms

EpS showed the highest EA (*p* < 0.01) across all three DNAm clocks compared to those generated from *SMARCA4-*deficient ATRT, *SMARCA4*-deficient MRT, and small cell carcinoma of the ovary, hypercalcemic type (SCCOHT) (supplementary 7, Fig. 2). EAA could not be computed as the age of patients was not available.

## Discussion

Here, we identified epigenetic age acceleration as a distinctive feature of epithelioid sarcoma using different types of epigenetic clocks and data sets. The reasons for this observation are unclear and need to be fully elucidated in larger cohorts and conducting functional experiments. Nevertheless, the following remarks and hypotheses can be derived from our observations.

Firstly, our observation points towards a marked perturbation of the aging process in EpS. Secondly, demonstrating marked differences between the methylation profiles of tumors with those of matched blood from two patients argues that the mechanism underlying the epigenetic age acceleration in EpS is unlikely to be caused by a systemic driver. Thirdly, considering the differences in the methylation profiles observed between *SMARCB1*- and *SMARCA4*-deficient neoplasms, the dysregulation of the PRC2 complex subsequent to the loss of inhibition by the SWI/SNF (*SMARCB1*- or *SMARCA4* loss) does not appear to account solely for the epigenetic acceleration observed in EpS. Fourthly, although still in its infancy, the idea of targeting aging pathways as a treatment for cancer is gaining traction [25–27]. Hence, given the intimate link between aging and cancer, the marked acceleration of aging in EpS could therefore constitute a therapeutic vulnerability for this generally aggressive disease for which new treatments are needed to improve survival. Examples of such targetable aging pathways in cancer include the mammalian target of rapamycin (mTOR), Sirtuins, AMPK (AMP-activated protein kinase), and PRC2 [28–31]. Targeting senescence in cancer represents another approach by selectively eliminating tumor cells using senolytics [32, 33]. In EpS specifically, it is noteworthy that inhibition of Enhancer of Zeste (EZH2), an enzymatic catalytic subunit of PRC2, by tazemetostat has shown promising clinical responses in patients with advanced EpS [34]. In addition, targeting the mTOR pathway with mTOR inhibitors abrogates EpS growth in pre-clinical models [35]. Together, the above evidence points to a global dysregulation of the pathways involved in aging in EpS. Finally, this study is, to the best of our knowledge, the first to provide EA and EAA in a large cohort of mesenchymal neoplasms. It is interesting to note that a substantial number of the subtypes investigated in the DKFZ reference data set of mesenchymal tumors are—as opposed to most cancers of epithelial lineage—characterized by negative epigenetic age acceleration [2].

In conclusion, our study reveals epigenetic age acceleration as a hallmark of EpS and highlights the potential of targeting aging pathways as an innovative treatment avenue for this rare sarcoma. In addition, as methylome profiles are increasingly used in routine clinical settings, EA and EAA scores can easily be generated and potentially used as diagnostic markers. Although our novel findings are based on a small number of samples, we hope that these will stimulate more research into this rare cancer.

### Supplementary Information

Below is the link to the electronic supplementary material.

Supplementary file1 (XLSX 9 KB)

Supplementary file2 (DOCX 7 KB)

Supplementary file3 (XLSX 18 KB)

Supplementary file4 (XLSX 88 KB)

Supplementary file5 (PDF 324 KB)

Supplementary file6 (XLSX 13 KB)

Supplementary file7 (PDF 346 KB)

## Data Availability

The raw data that support the findings of this study are available in the European Genome-phenome Archive (EGA).

## Abbreviations

*DNAm*: DNA methylation
*CA*: Chronological age
*PRC2*: Polycomb repressive complex 2
*SWI/SNF*: Chromatin remodeling complex SWItch/Sucrose Non-Fermentable
*EpS*: Epithelioid sarcoma
*ATRT*: Atypical teratoid and rhabdoid tumor
*SCCOHT*: Small cell carcinoma of the ovary, hypercalcemic type
*MRT*: Malignant rhabdoid tumor
*RNOH*: Royal National Orthopaedic Hospital
*ROH*: Royal Orthopaedic Hospital
*EA*: Epigenetic age
*EAA*: Epigenetic age acceleration
*FF*: Fresh-frozen
*EZH2*: Enhancer Of Zeste 2
*mTOR*: Mammalian target of rapamycin
