# Supplementary material for: Epigenetic age acceleration is a distinctive trait of epithelioid sarcoma with potential therapeutic implications
Source: GeroScience. 2024 Jun 16;46(5):5203–9. doi: 10.1007/s11357-024-01156-6 (PMC11336154; doi:10.1007/s11357-024-01156-6)
Supplement: Supplementary file 2 — Supplementary file2 (DOCX 7 KB) [file 11357_2024_1156_MOESM2_ESM.docx]

**Supplementary 2., DNA extraction protocol**

Tumour DNA was extracted from 10 μM sections of snap frozen fresh samples using a QIAamp DNA Mini kit (Qiagen, Germany) using the standard kit protocol and eluted in the kit elution buffer. The samples were incubated overnight, and additional proteinase K added (up to 20µL) if the tissue had not fully lysed. All DNA samples were quality controlled using NanoDrop™ 1000 (Thermofisher Scientific, USA), Qubit™ dsDNA HS (Thermofisher Scientific, USA) and Genomic DNA ScreenTape Assay (Agilent technologies, USA). Samples of sufficient purity (A280/260: 1.8-2), concentration (>20ng/µL) and integrity (DIN score >6) were sent for analysis. Tumour RNA was extracted from 10 µM sections of the same frozen sample from which the DNA was extracted. The sections were lysed in TRIzol® (Thermofisher Scientific, USA) using the Zymo Direct-zol RNA Miniprep kit (Cambridge Scientific, UK). The TRIzol® reagent was phase separated with chloroform, and the aqueous phase run through the standard Zymo kit protocol with a 15-minute DNase step. The RNA was eluted in nuclease-free water. Quality assessment was undertaken using the NanoDrop™ 1000 and RNA ScreenTape Assay (Agilent technologies, USA). Samples of sufficient integrity (RIN score >6) and yield (>1µg) were submitted for analysis.
