# Supplementary figures and images for: Epigenetic age acceleration is a distinctive trait of epithelioid sarcoma with potential therapeutic implications
Source: GeroScience. 2024 Jun 16;46(5):5203–9. doi: 10.1007/s11357-024-01156-6 (PMC11336154; doi:10.1007/s11357-024-01156-6)

A

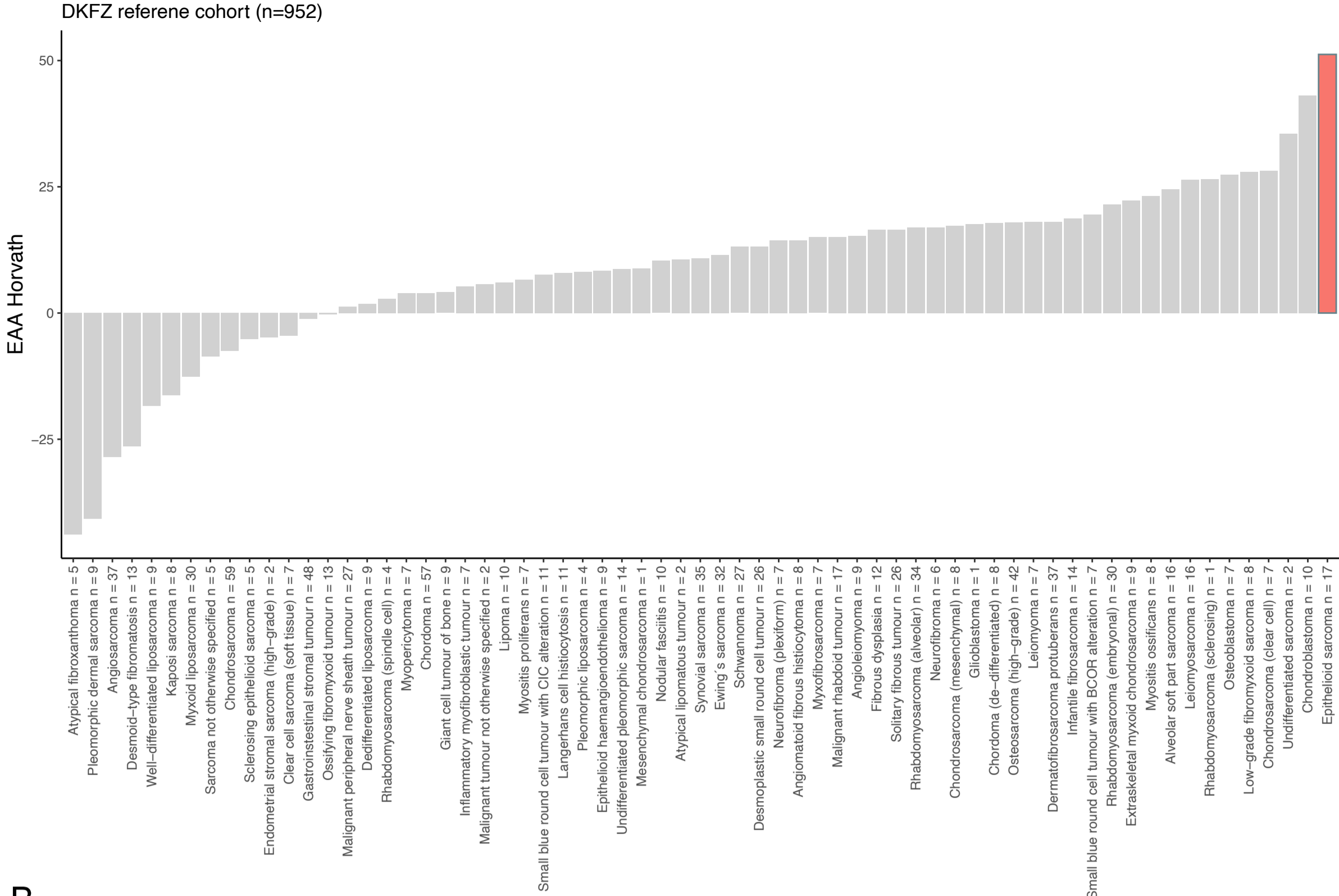

B

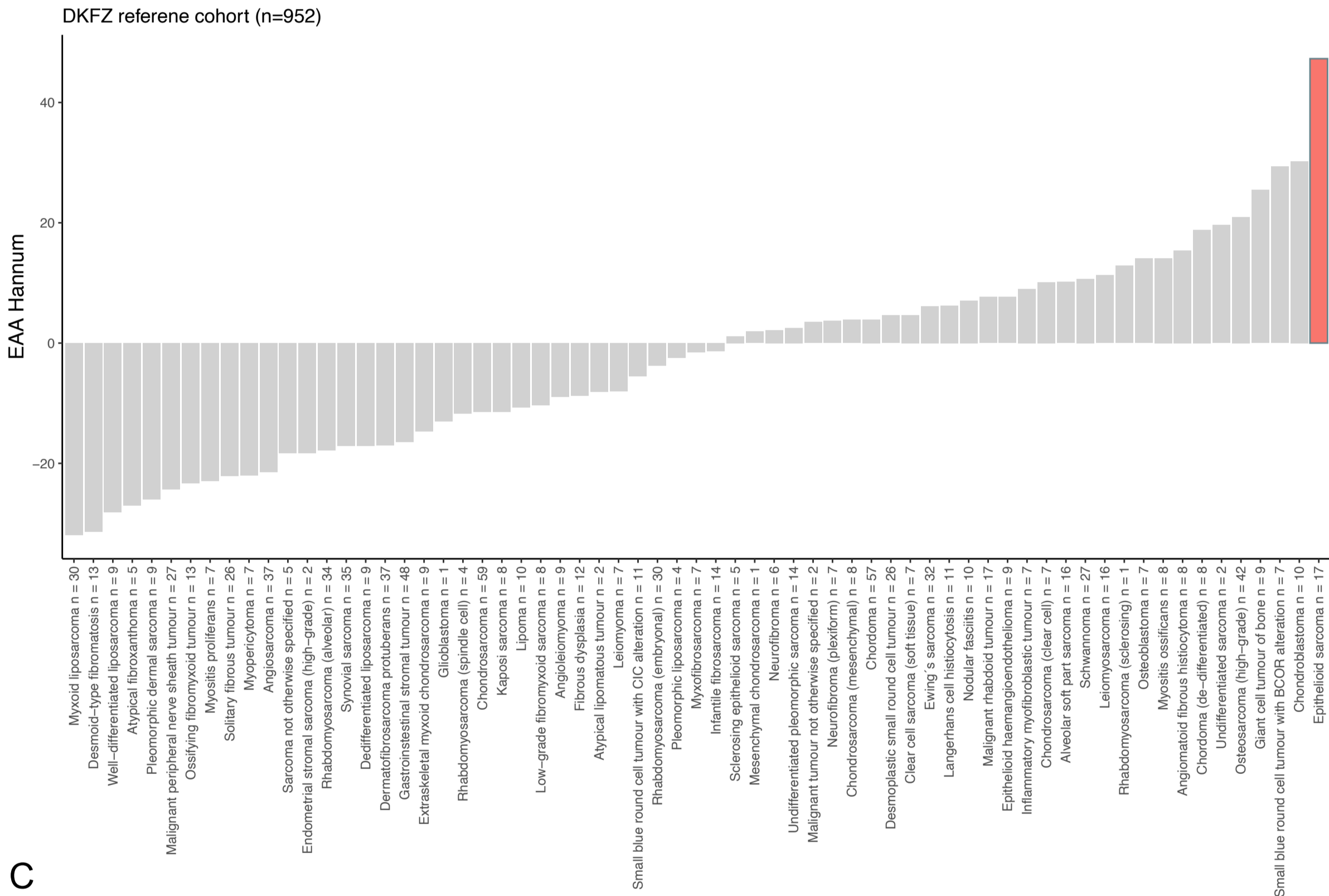

C

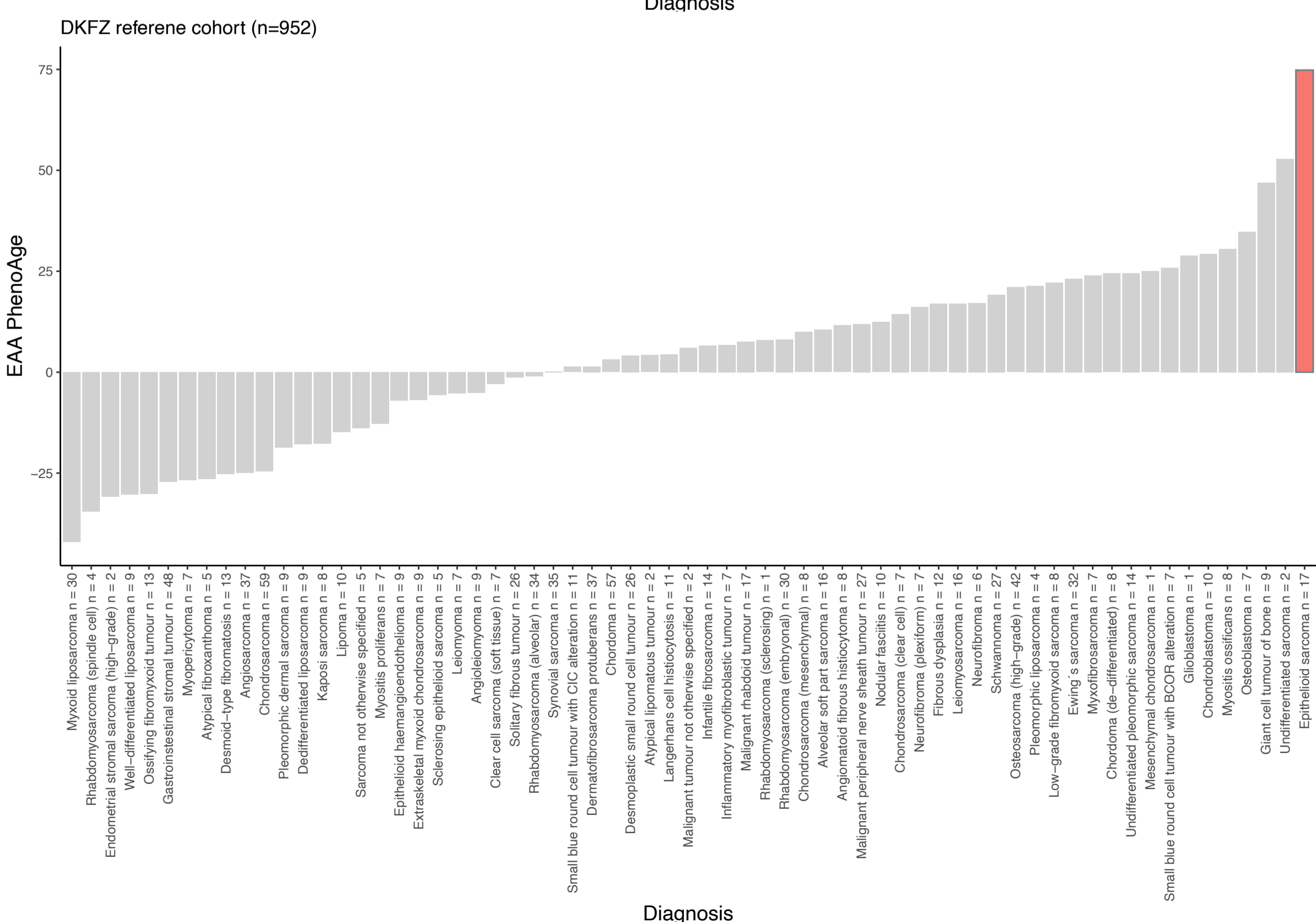

Supplement: Supplementary file 5 — Supplementary file5 (PDF 324 KB) [file 11357_2024_1156_MOESM5_ESM.pdf]
