# Supplementary material for: Epigenetic age acceleration is a distinctive trait of epithelioid sarcoma with potential therapeutic implications
Source: GeroScience. 2024 Jun 16;46(5):5203–9. doi: 10.1007/s11357-024-01156-6 (PMC11336154; doi:10.1007/s11357-024-01156-6)
Supplement: Supplementary file 7 — Supplementary file7 (PDF 346 KB) [file 11357_2024_1156_MOESM7_ESM.pdf]

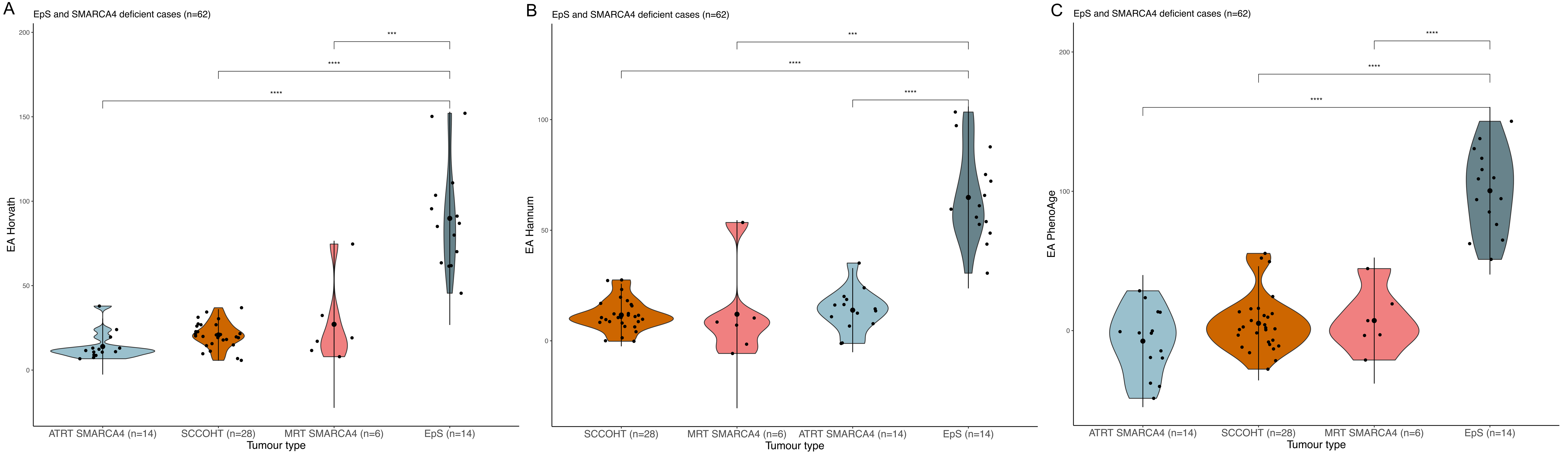

A
EpS and SMARCA4 deficient cases (n=62)
EA Horvath
Tumour type
ATRT SMARCA4 (n=14)
SCCOHT (n=28)
MRT SMARCA4 (n=6)
EpS (n=14)
B
EpS and SMARCA4 deficient cases (n=62)
EA Hannum
Tumour type
SCCOHT (n=28)
MRT SMARCA4 (n=6)
ATRT SMARCA4 (n=14)
EpS (n=14)
C
EpS and SMARCA4 deficient cases (n=62)
EA PhenoAge
Tumour type
ATRT SMARCA4 (n=14)
SCCOHT (n=28)
MRT SMARCA4 (n=6)
EpS (n=14)
